# Vitamin C in Complex Regional Pain Syndrome in Patients with Distal Radius Fracture

**DOI:** 10.1055/s-0045-1810402

**Published:** 2025-08-25

**Authors:** Fernando Antonio Silva de Azevedo Filho, Roberta Muniz Blunck, Abdouni Y Ali, Patrícia Maria de Moraes Barros Fucs, Ricardo Britto Cotias

**Affiliations:** 1 Hospital do Subúrbio, Salvador, Bahia, Brazil; 2 Faculdade de Ciências Médicas, Santa Casa de São Paulo, São Paulo, SP, Brazil; 3 Irmandade da Santa Casa de Misericórdia de São Paulo, São Paulo, SP, Brazil

**Keywords:** ascorbic acid, chronic pain, complex regional pain syndromes, radius fractures, ácido ascórbico, dor crônica, fraturas do rádio, síndrome da dor regional complexa

## Abstract

**Objective:**

To evaluate the effect of vitamin C in preventing complex regional pain syndrome (CRPS) in patients with fracture of the distal end of the radius (FDER).

**Methods:**

The present study included FDER patients with an indication for surgical treatment and aged over 18 years. We evaluated the age group, gender, dominant side, presence of comorbidities, affected side, trauma mechanism, and the surgical technique. Participants were randomized into 2 groups: placebo ( *n* = 64), which received microcrystalline cellulose, and intervention ( *n* = 58), treated with 1 g/day of vitamin C in a single dose for 60 days, starting immediately after surgery.

**Results:**

The average incidence of neuropathic pain was 17% higher in the placebo group than in the vitamin C one during the evaluation period ( *p* = 0.008). The average visual analog scale (VAS) for pain was 1.22 points higher in the placebo group regardless of evaluation moment ( *p* = 0.001). The CRPS at 12 and 24 weeks were statistically more frequent in the placebo group ( *p* = 0.014 and 0.007, respectively).

**Conclusion:**

Vitamin C has an effect on preventing neuropathic pain and CRPS, as well as on controlling postoperative pain control.

## Resumo

**Objetivo:**

Avaliar o efeito da vitamina C na prevenção da síndrome da dor complexa regional (SDCR) em pacientes com fratura da extremidade distal do rádio (FEDR).

**Métodos:**

Foram incluídos pacientes com FEDR com indicação de tratamento cirúrgico e idade acima de 18 anos, sendo avaliados faixa etária, gênero, dominância, presença de comorbidades, lado acometido, mecanismo do trauma e técnica cirúrgica. Os participantes foram randomizados em dois grupos: placebo ( *N* = 64), que recebeu celulose microcristalina, e intervenção ( *N* = 58) , que recebeu 1 g/dia de vitamina C, em dose única durante 60 dias, iniciando no pós-operatório imediato.

**Resultados:**

A incidência de dor neuropática foi em média 17% maior no grupo placebo do que no grupo vitamina C nas semanas avaliadas ( *p* = 0,008). A escala visual análoga (EVA) foi em média 1,22 pontos maior no grupo placebo independente do momento de avaliação ( *p* = 0,001). A SDCR, em 12 e 24 semanas, foi estatisticamente mais frequente no grupo placebo ( *p* = 0,014 e 0,007 respectivamente).

**Conclusão:**

A vitamina C tem efeito na prevenção da dor neuropática e da SDCR, bem como no controle da dor pós-operatória.

## Introduction

Distal radius fractures (DRFs) are the most common fractures. They present a clear bimodal pattern in children under 15 and adults over 50, who are at increased risk of fracture. Treatment includes closed reduction and cast immobilization or surgery. 1

In the immediate postoperative period, direct nociceptor activation in the inflammatory response and potential injury to nerve structures clinically presents with pain during rest at the surgical site and adjacent regions, in addition to pain triggered by touch or movement, indicating peripheral sensitization. 2

Complex regional pain syndrome (CRPS) is a complication featuring chronic and persistent pain with no cellular damage. It presents as autonomic and sensory pain, and motor, trophic, and vasomotor abnormalities leading to limb dysfunction. 3 4 The incidence of CRPS in patients with DRF is approximately 4%. 4 Some studies reported incidences from 1 to 37%, with a direct impact on quality of life, well-being, and work capacity. 5 6 Its clinical presentation varies from type I to II, respectively without or with evidence of nerve damage. 3 4

Several recent clinical studies have shown that vitamin C administration to patients with CRPS, acute and post-herpetic neuralgia, and cancer-related pain significantly results in pain relief and improves the quality of life. It is also a powerful antioxidant that neutralizes free radicals and reduces oxidative stress, potentially contributing to treating inflammation and chronic pain.. It has proven safe and effective in relieving acute and chronic pain, including in outpatient, surgical, and oncology patients, and may decrease opioid use. 7 8

Vitamin C plays a complex and multifaceted role in pain modulation, primarily through its antioxidant properties, regulation of inflammation, and support of connective tissue health. However, further research is required to fully elucidate its direct influence on pain and define specific guidelines for its clinical application.

The present study aimed to evaluate vitamin C's efficacy in preventing neuropathic pain and CRPS onset in patients with DRF undergoing surgical treatment and assess its impact on postoperative pain control.

## Materials and Methods

This randomized, double-blind clinical study included patients with DRF undergoing surgical treatment at the orthopedics and traumatology service of a tertiary hospital.

The sample included patients over 18 years old admitted from April 2023 to April 2024 with DRF and indication for surgical treatment. We excluded pediatric subjects, polytraumatized patients, bilateral fractures, subjects with chronic renal failure, a history of kidney stones, ipsilateral DRF, diagnosis of neuropathic pain, history of glucose-6-phosphate dehydrogenase deficiency and hyperoxaluria, vitamin C allergy, under vitamin C or multivitamin treatment, or not agreeing to participate in the research protocol.

We evaluated the following clinical aspects: age range, gender, dominant side, presence of comorbidities, affected side, trauma mechanism, and surgical technique used. The radiographic evaluation consisted of images in anteroposterior and lateral views of the injured wrist submitted to the Arbeitsgemeinschaft für Osteosynthesefragen (AO) classification.

An online research tool ( https://www.randomizer.org/ ) was used to randomize the participants into two groups. The placebo group ( *n* = 64) received microcrystalline cellulose, and the intervention one ( *n* = 58) received 1 g/day of vitamin C 9 in a single dose for 60 days starting immediately after surgery. A medical team member prescribed the medication, without informing the patient and the main researcher. The latter was did the 24-week monitoring.

All patients followed the same analgesia protocol, with dipyrone 1 g every 6 hours as the first choice of common analgesic agent and, in case of allergy, paracetamol 750 mg every 6 hours with a weak opioid. For the opioids, the first choice was tramadol 100 mg every 12 hours and, for allergic patients, codeine 30 mg was the second one. The patients received instructions to take the medications based on pain intensity.

At admission, the Quick Disabilities of the Arm, Shoulder, and Hand (Quick-DASH, *d* ) questionnaire assessed upper limb functionality. 10 This tool consists of 11 questions to assess physical function during the last week. Its score ranges from 0 to 5 for each item, and the final score ranges from 0 to 100. Furthermore, all patients underwent an evaluation for depression and anxiety using the Hospital Anxiety and Depression Scale (HADS). 11

We performed clinical, functional, and radiographic evaluations at five points, at 2, 4, 6, 12, and 24 weeks. The patient, the attending surgeon, and the main researcher were blinded to randomization, with the last one being responsible for clinical follow-up and radiological fracture healing assessment.

At each follow-up appointment, we asked patients to assess their pain intensity at that moment using a visual analog scale (VAS), a one-dimensional tool with a line ranging from 0 to 10 points, indicating “no pain” and “worst pain imaginable”, respectively. We also assessed the presence of treatment-related complications and CRPS signs and symptoms using the Budapest criteria. 12

At the 12 ^th^ -week evaluation, we defined the presence of neuropathic pain with the *Douleur neuropathique 4* (DN4) questionnaire, consisting of seven items referring to symptoms and three to the physical examination. Each item scores one for positive answers and zero for negative answers. A total score equal to or greater than four suggests neuropathic pain. 13

In the 24 ^th^ week of follow-up, patients underwent a new clinical evaluation using the same tools along with VAS for pain quantification.

Those diagnosed with CRPS underwent drug treatment with pregabalin or gabapentin, continued the follow-up in the service, and received a referral for monitoring by a physical therapy team.

The Excel 2013 (Microsoft Corp.) software stored the collected data, while the analysis used the Statistical Package Social Sciences (SPSS, IBM Corp.) for Windows, version 22.0. The significance level of the tests was 5%.

The description of qualitative characteristics used absolute and relative frequencies, while chi-square or exact tests (Fisher's or likelihood ratio) determined any association. The description of quantitative characteristics used summary measures (mean, standard deviation [SD], median, and quartile values), and the unpaired Student's t-test or Mann-Whitney test performed group comparisons. The presentation of VAS scores and the presence of neuropathic pain at the five points relied on summary measures, absolute and relative frequencies, respectively compared groups and times using generalized estimating equations (GEEs) with normal distribution, identity link function, binomial distribution, and logical link function, assuming a first-order autoregressive correlation matrix (AR[1]) between the evaluation moments of the same patient. Next, Bonferroni's multiple comparisons revealed differences between groups and times.

The Research Ethics Committee evaluated and approved this study under number CAAE 63013922.1.0000.0040.

## Results

The sample consisted of 137 patients, of whom 122 completed the 24-week follow-up, including 55 women (45.1%) and 67 men (54.9%) with an average age of 48.1 years. Table 1 details the characteristics of each group.

**Table 1:** Description of personal and clinical characteristics according to groups and statistical test results

Variable	Group		Total ( *N* = 122)	*p* -value
	Placebo ( *N* = 64)	Vitamin C ( *N* = 58)		
**Age (years)**				
mean ± SD	47.9 ± 15.4	48.3 ± 15.5	48.1 ± 15.4	0.909**
median (25p; 75p)	47 (37.3; 63.8)	48.5 (36.8; 60.8)	47.5 (37; 63)	
**Gender, n (%)**				
Female	29 (45.3)	26 (44.8)	55 (45.1)	0.957
Male	35 (54.7)	32 (55.2)	67 (54.9)	
**Dominant side, n (%)**				
Right	62 (96.9)	54 (93.1)	116 (95.1)	0.422*
Left	2 (3.1)	4 (6.9)	6 (4.9)	
**Comorbidities, n (%)**				
None	46 (71.9)	40 (69)	86 (70.5)	0.682#
SAH	6 (9.4)	7 (12.1)	13 (10.7)	
DM	1 (1.6)	4 (6.9)	5 (4.1)	
SAH + DM	5 (7.8)	3 (5.2)	8 (6.6)	
Psychiatric disorders	2 (3.1)	1 (1.7)	3 (2.5)	
Other	4 (6.3)	3 (5.2)	7 (5.7)	
**Injured side, n (%)**				
Right	33 (51.6)	22 (37.9)	55 (45.1)	0.131
Left	31 (48.4)	36 (62.1)	67 (54.9)	
**Trauma mechanism, n (%)**				
Low energy	27 (42.2)	29 (50)	56 (45.9)	0.387
High energy	37 (57.8)	29 (50)	66 (54.1)	
**AO, n (%)**				
Extra-articular	31 (48.4)	13 (22.4)	44 (36.1)	**0.008**
Partial articular	6 (9.4)	5 (8.6)	11 (9)	
Articular	27 (42.2)	40 (69)	67 (54.9)	
**Treatment, n (%)**				
Closed reduction (Kirschner wire)	45 (70.3)	34 (58.6)	79 (64.8)	0.177
ORIF	19 (29.7)	24 (41.4)	43 (35.2)	
**HADS**				
mean ± SD	8.1 ± 6.6	7.7 ± 6.4	7.9 ± 6.5	0.768£
median (25p; 75p)	7 (3; 12)	7 (3; 10)	7 (3; 11)	

**Abbreviations:** AO, AO/OTA classification; DM, diabetes mellitus; HADS, hospital anxiety and depression scale; ORIF, open reduction and internal fixation; SAH, systemic arterial hypertension; SD, standard deviation. **Notes:** Chi-square test; *, Fisher's exact test; #, Likelihood ratio test; **Unpaired Student's t-test; £, Mann-Whitney test.

The behavior of the groups was statistically similar for neuropathic pain and VAS throughout the evaluation (p _Interaction_ > 0.05). Neuropathic pain presented statistical differences between groups at all times (p _Group_ = 0.013). Moreover, the average VAS differed between groups regardless of the evaluation moment (p _Group_ = 0.001) and throughout time in both groups (p _Time_ < 0.001), as shown in Table 2 .

**Table 2:** Description of neuropathic pain and VAS for pain according to groups, evaluation times, and result comparisons

Variable	Group		p _Group_	p _time_	p _interaction_
	Placebo ( *N* = 64)	Vitamin C ( *N* = 58)			
**Neuropathic pain**			**0.013**	0.091	0.355
**12 weeks**					
DN4 <4	45 (70.3)	50 (86.2)			
DN4 ≥4	19 (29.7)	8 (13.8)			
**24 weeks**					
DN4 <4	47 (73.4)	53 (91.4)			
DN4 ≥4	17 (26.6)	5 (8.6)			
**VAS**			**0.001**	**<0.001**	0.147
**2 weeks**					
mean ± SD	3.9 ± 3.1	2.8 ± 2.4			
median (25p; 75p)	5 (0; 7)	2 (1; 5)			
**4 weeks**					
mean ± SD	3.5 ± 2.9	1.7 ± 2.2			
median (25p; 75p)	3 (0; 6)	1 (0; 3)			
**6 weeks**					
mean ± SD	3 ± 3.2	1.4 ± 2.1			
median (25p; 75p)	2 (0; 6)	0 (0; 2)			
**12 weeks**					
mean ± SD	2.1 ± 2.9	1.3 ± 2			
median (25p; 75p)	0 (0; 3.8)	0 (0; 2)			
**24 weeks**					
mean ± SD	1.8 ± 2.8	0.9 ± 1.6			
median (25p; 75p)	0 (0; 2)	0 (0; 2)			

**Abbreviations:** AR(1), first-order autoregressive correlation matrix; DN4, *Douleur neuropathique 4* questionnaire; GEE, generalized estimating equation; SD, standard deviation; VAS, visual analog scale. **Notes:** GEEs with binomial distribution and logit link function; GEEs with normal distribution and identity link function; both models assumed an AR(1) correlation matrix between times.

The frequency of neuropathic pain was on average 17% higher in the placebo group than in the vitamin C group during the study period ( *p* = 0.008). The average VAS was 1.22 points higher in the placebo group regardless of the evaluation time ( *p* = 0.001) and presented a statistically significant decrease throughout the weeks in both groups ( *p* < 0.05). However, no statistically significant differences occurred between consecutive evaluations, i.e., 4 to 6 weeks, 6 to 12 weeks, and 12 to 24 weeks ( *p* > 0.05), as can be seen in Table 3 .

**Table 3:** Neuropathic pain e EVA results from multiple comparisons between groups and times

Variable	Comparison		Men difference	SE	*p* -value	95% CI	
						Inferior	Superior
Neuropathic pain (%)	Placebo and Vitamin C		17.0	6.4	**0.008**	5.0	30.0
VAS	Placebo and Vitamin C		1.22	0.37	**0.001**	0.50	1.94
	2–4 weeks		0.76	0.18	**<0.001**	0.25	1.28
	2–6 weeks		1.20	0.24	**<0.001**	0.53	1.87
	2–12 weeks		1.71	0.27	**<0.001**	0.95	2.47
	2–24 weeks		2.08	0.29	**<0.001**	1.26	2.90
	4–6 weeks		0.44	0.18	0.175	−0.08	0.95
	4–12 weeks		0.94	0.24	**0.001**	0.27	1.62
	4–24 weeks		1.31	0.27	**<0.001**	0.55	2.08
	6–12 weeks		0.51	0.18	0.059	−0.01	1.02
	6–24 weeks		0.88	0.24	**0.002**	0.21	1.55
	12–24 weeks		0.37	0.18	0.443	−0.15	0.89

**Abbreviations:** CI, confidence interval; SE, standard error; VAS, visual analog scale. **Notes:** Bonferroni's multiple comparisons.

In the 12 ^th^ and 24 ^th^ weeks, CRPS was statistically more frequent in the placebo group ( *p* = 0.014 and 0.007, respectively). The Quick-DASH score at 24 weeks was statistically lower in the vitamin C than in the placebo group ( *p* = 0.040), as shown in Table 4 .

**Table 4:** Statistical analysis results of CRPS and Quick-DASH according to groups at different assessment times

Variable	Group		Total ( *N* = 122)	*p* -value
	Placebo ( *N* = 4)	Vitamin C ( *N* = 58)		
**CRPS at 12 weeks**				
No	57 (89.1)	58 (100)	115 (94.3)	0.014*
Yes	7 (10.9)	0 (0)	7 (5.7)	
**CRPS at 24 weeks**				
No	56 (87.5)	58 (100)	114 (93.4)	0.007*
Yes	8 (12.5)	0 (0)	8 (6.6)	
**Quick-DASH at 24 weeks**				
mean ± SD	15.2 ± 25.5	5.9 ± 13	10.8 ± 21	**0.040£**
median (25p; 75p)	0 (0; 25)	0 (0; 0)	0 (0; 16)	

**Abbreviations:** CRPS, complex regional pain syndrome; Quick-DASH, quick disabilities of the arm, shoulder and hand; SD, standard deviation. **Notes:** *Fisher's exact test; #Likelihood ratio test; **Unpaired Student's t-test; £Mann-Whitney test.

Fracture severity per AO classification had no statistically significant association with CRPS and neuropathic pain ( *p* > 0.05).

Patients with neuropathic pain at 12 weeks had a statistically higher HADS score ( *p* = 0.048), with a similar difference in the 24 weeks without statistical significance ( *p* = 0.220).

## Discussion

Patients treated with vitamin C had superior postoperative pain control compared with those receiving placebo. This supplementation was shown to significantly reduce pain intensity, which may be attributed to its effect on modulating inflammation and reducing oxidative stress. These results suggest that vitamin C can be an effective intervention for improving postoperative pain management, which is consistent with the literature.

Evidence suggests that ascorbic acid (vitamin C) administration may have analgesic properties in some clinical conditions. 7 This is an essential micronutrient with evidence supporting its role in several metabolic processes related to mental health, stress response, bone formation, tissue repair, collagen production, and pain perception. 8 14 It influences the inflammatory response, potentially modulating the production of inflammatory cytokines and the activity of immune cells, reducing pain-associated inflammation. 7

Vitamin C is a water-soluble antioxidant with high levels in the central nervous system that exceed serum concentrations by 10-fold. 15 16 17 It is a powerful antioxidant and anti-inflammatory agent, constituting a cofactor for adrenal steroidogenesis and catecholamine biosynthesis. It can also increase endomorphin and endorphin synthesis, and it is a cofactor for the biosynthesis of amidated opioid peptides. 7 8

Evidence suggests that N-methyl-D-aspartate (NMDA) receptors largely mediate the nociceptive response of ascorbic acid, specifically by interacting with ionotropic glutaminergic receptors. There is good evidence regarding the involvement of NMDA receptors in pain modulation, as they reduce transmission and exert antinociceptive action. 15 17 18

As described in a review by Fukushima and Yamazaki, the increased demand for ascorbic acid in surgical settings potentially results from oxidative stress. Therefore, postoperative patients require daily doses higher than those recommended, and the administration of exogenous vitamin C is associated with better surgical outcomes. 19 Studies showed that its administration is also associated with a reduced need for postoperative opioid analgesics. 20

The significant positive impact of vitamin C in preventing neuropathic pain and CRPS onset presented here is consistent with the literature. Patients receiving it showed a notable reduction in the incidence and intensity of neuropathic pain and a lower occurrence of CRPS compared with the placebo group. These effects may be attributed to the ability of vitamin C to reduce inflammation and oxidative stress, critical factors in the development and maintenance of these painful conditions.

Free radicals play a critical role in generating pain in several diseases, including neuropathic and inflammatory conditions. Antioxidants may participate in modulation inhibition and attenuate injury-induced mechanical allodynia. Their combination has a greater antiallodynic effect on neuropathic pain processing in the spinal cord. 21

Studies revealed that ascorbic acid protects cortical neurons from the toxic effects of NMDS mediated by its receptors. 15 Brain ascorbic acid levels have an association with the activity of these receptors and increasing their concentration may benefit patients with risk for neurological complications. 15 17 22 23

Randomized clinical trials investigated the effects of vitamin C on CRPS incidence in patients undergoing wrist and ankle surgery. In these studies, the doses ranged from 0.2 to 1.5 g/day for 45 to 50 days after surgery. Its administration resulted in lower CRPS incidences, with doses ≥ 0.5 g/day as the most effective. 9 24 25 26 Previous research has indicated surgical patients require supplementation doses higher than 0.5 g/day to restore their normal status. 7 19

Vitamin C supplementation has been associated with improved functional outcomes, including decreased pain and risk of CRPS after orthopedic surgery. 27

Contrary to our results and the literature data previously described, Ekrol et al. 24 found no significant difference in the DASH score, CRPS incidence, or fracture consolidation over 1 year, concluding that vitamin C administration has no benefit for patients with DRF.

A meta-analysis of seven randomized controlled trials demonstrated that oral vitamin C supplementation can reduce the risk of CRPS type I but did not improve functional outcomes in orthopedic patients. 28

One limitation of this study was the number of participants. Although the minimum sample size was determined to be 850 individuals, we obtained promising results with 122. This can be attributed to a more pronounced therapeutic effect than previously considered, to the intra- and intergroups variability, or to the high adherence to the intervention protocol. In some situations, a significant clinical difference between groups can translate into statistical significance even with a smaller sample size. It is worth highlighting that despite the promising results, this limitation may have impacted on the robustness and generalizability of the findings. As such, studies a larger sample size are recommended for confirmation and external validation.

## Conclusion

The present study demonstrated that vitamin C can have a positive influence on the prevention of neuropathic pain, CRPS, as well as control of postoperative pain and inflammation in patients with DRF.
